# Soluble Receptor for Advanced Glycation End Product Is Involved in the Inflammatory Response of Human Adenovirus-Infected Patients

**DOI:** 10.3389/fmicb.2022.923215

**Published:** 2022-07-07

**Authors:** Wen Xu, Cheng-Jun Wu, Yan-Mei Jiao, Xiao-Le Mei, Lei Huang, En-Qiang Qin, Bo Tu, Peng Zhao, Li-Feng Wang, Wei-Wei Chen

**Affiliations:** ^1^Senior Department of Infectious Diseases, The Fifth Medical Center of PLA General Hospital, National Clinical Research Center for Infectious Diseases, Beijing, China; ^2^IC Technology Key Lab of Liaoning, School of Biomedical Engineering, Dalian University of Technology, Dalian, China

**Keywords:** sRAGE, human adenovirus, pneumonia, inflammation, MAPK

## Abstract

Human adenovirus (HAdV) infection causes excessive inflammation associated with severe tissue injury, such as pneumonia. The molecules involved in the underlying inflammatory mechanisms remain to be elucidated. Receptor for advanced glycation end product (RAGE) is mainly expressed on immune cells and lung tissues, and it is a key factor in the initiation and development of inflammation. RAGE can be cleaved by metalloprotease 9 (MMP9) to release the extracellular segment, which is named soluble RAGE (sRAGE), into the intercellular space, where it can bind to RAGE ligands and block RAGE activation and subsequent inflammation. In our study, we enrolled HAdV-infected patients and their contacts to examine the relationship between sRAGE and inflammation induced by HAdV infection. The results showed that HAdV infection stimulated inflammatory cytokine secretion, increased such as high mobility group box 1 (HMGB1) levels, and suppressed sRAGE expression. sRAGE levels were significantly different between patients with or without pneumonia. We also found that MMP9 was significantly lower in patients with pneumonia, and it was positively correlated with sRAGE levels over 7 days after disease onset. The mitogen-activated protein kinase (MAPK) pathway is an important immune activation signaling pathway that is regulated by RAGE. We observed the activation of the MAPK pathway in the peripheral blood mononuclear cells (PBMCs) of patients. Negative correlations between sRAGE and phosphorylated JNK and p38 were observed. These results suggest that sRAGE is involved in HAdV-induced inflammatory responses, and might be a potential therapeutic target to alleviate the HAdV-induced excessive inflammation.

## Introduction

Human adenoviruses (HAdVs) belong to the genus Mastadenovirus of the family Adenoviridae. About 100 distinct genotypes have been identified to date, and these genotypes can be divided into 7 species (A–G) (Lynch and Kajon, 2021). HAdV infection typically causes asymptomatic to mild infections involving the upper or lower respiratory tract, gastrointestinal tract, or conjunctiva, and most cases are self-limited. However, HAdV can cause severe/fatal pneumonia in both immunocompetent and immunocompromised individuals (Lynch and Kajon, 2021). Kindergarten children, students, and military recruits are very vulnerable to HAdV infection (Lynch and Kajon, 2021). In immunocompromised patients, especially in patients undergoing transplantation, HAdV may cause severe symptoms, such as hepatitis, nephritis, meningoencephalitis, and pneumonia, and can have a mortality rate of up to 70%, especially in pediatric patients undergoing stem cell transplantation (Lion, 2014). HAdV also causes severe outcomes in previously healthy adults and children (Chuang et al., 2003; Moura et al., 2007; Bhatti and Dhamoon, 2017). To date, no specific treatments are available for severe HAdV infections (Lynch and Kajon, 2021).

Human adenoviruse is the prevalent agent in patients with an acute respiratory infection and causes pneumonia worldwide (Wen et al., 2021). Excessive inflammatory responses in patients infected by HAdV are thought to be critical in immunopathological tissue injury in adenoviral pneumonia (Kajon et al., 2003). Recent research revealed that excessive inflammatory T-cell responses and cytokine overproduction are linked to the development of severe outcomes after adenovirus infection (Chen et al., 2014). CD8 T-cell responses also contribute to pulmonary inflammation during adenovirus infection (Molloy et al., 2017). More efforts are needed to elucidate the underlying mechanism of HAdV-induced inflammation.

Receptor for advanced glycation end products (RAGE) is a transmembrane protein in the immunoglobulin superfamily that is involved in the initiation and persistence of inflammation (Hudson and Lippman, 2018). RAGE is constitutively expressed in lung tissue and immune cells (Bopp et al., 2008). It binds a number of diverse endogenous ligands [such as high mobility group box 1 (HMGB1), S100A12, S100B, advanced glycation end-products] and exogenous ligands [such as LPS, CpG DNA, and respiratory syncytial virus (RSV) F protein] (Hudson and Lippman, 2018). RAGE pathway activation promotes the formation of reactive oxygen species (ROS) and the activation of mitogen-activated protein kinase (MAPK) and NF-κB (Oczypok et al., 2017). Single nucleotide polymorphisms (SNPs) of RAGE genes are associated with increased acute respiratory distress syndrome (ARDS) risk, suggesting that the RAGE pathway is involved in the underlying mechanism of severe lung injury (Jabaudon et al., 2018). During influenza A virus infection, the RAGE pathway is responsible for cytokine production (van Zoelen et al., 2009). Increased RAGE expression in lung tissue is associated with pneumonia (Morbini et al., 2006). RAGE-deficient mice were protected from RSV and influenza virus infection-induced inflammation (van Zoelen et al., 2009; Miller et al., 2012). Blocking RAGE could also be a therapeutic strategy to alleviate excessive lung inflammation and injury (Blondonnet et al., 2017).

Moreover, the extracellular domain of RAGE is cleaved by the metalloproteinases ADAM10 and metalloprotease 9 (MMP9), and the extracellular segment of RAGE is released, forming soluble RAGE (sRAGE) (Metz et al., 2012). sRAGE acts as a decoy and binds to RAGE ligands, reducing RAGE activation (Zhang et al., 2021). These processes form an elegant negative-feedback loop to contain RAGE concentrations and activation, and control the extent of inflammatory responses. A multicenter observational study demonstrated that baseline sRAGE levels could be an independent predictor of ARDS (Jabaudon et al., 2018). sRAGE treatment prevented RSV-induced weight loss and reduced neutrophilic inflammation (Miller et al., 2012). Understanding the role of sRAGE in respiratory infection, particularly in clinical settings, will promote the development of treatments targeting sRAGE.

In this study, we enrolled HAdV-infected patients and their contacts, and the results showed that HAdV-infected patients had higher inflammatory cytokines and lower sRAGE levels than their contacts. sRAGE was significantly different between patients with or without pneumonia. sRAGE levels were associated with immune cell activation, as indicated by MAPK phosphorylation. The MMP9 expression was correlated with changes in sRAGE. Our results suggest that sRAGE is an important molecule involved in HAdV infection-induced inflammation, and is a potential therapeutic target for treating adenoviral pneumonia.

## Materials and Methods

### Study Population

The HAdV outbreaks occurred in Beijing (February to March 2018). Patients with obvious respiratory symptoms were treated in the 5th Medical Center, General Hospital of People’s Liberation Army. HAdV-infected patients and their contacts were enrolled in this study. The pathogen causing this outbreak was confirmed to be HAdV type 7 by sequencing the hexon, fiber, and penton genes.

The HAdV-infected patients were diagnosed if the HAdV genome was detected by PCR in throat swab samples and they had a fever >38°C. Patients were excluded if they were positive for circulating influenza or chlamydia pneumonia antibodies. The enrolled patients received supportive therapy and surveillance of fluid and electrolytes. Those who had inconveniently high fevers were given fever medications. Patients were discharged from the hospital when they met the following criteria: normal routine blood test, undetectable HAdV genome, and no obvious clinical symptoms.

Throat swabs and peripheral blood samples were collected before breakfast by skilled nurses. The samples were processed in approximately 2 h and stored at −80°C.

Informed consent was obtained from all enrolled subjects. The protocol for this study was reviewed and approved by Beijing 302 Hospital Research Ethics Committee (2018032D). This work was carried out in accordance with the Declaration of Helsinki.

### Detection of Cytokines, High Mobility Group Box 1, Soluble Receptor for Advanced Glycation End Product, and Metalloprotease 9 in Plasma

Peripheral inflammatory cytokines [interleukin 6 (IL-6), interferon gamma (IFN-γ), interferon alpha 1 (IFN-α1), IP-10, IL-10, HMGB1, sRAGE, and MMP9) were measured by commercial ELISA kits (Abcam, United States) according to the instructions provided by the manufacturer. Briefly, after being diluted to the desired ratio, Plasma samples, and standards were added to plates coated with specific antibodies. Then, detection antibodies tagged with HRP were added. After incubation, TMB substrate solution was added, and the reaction was stopped by stop solution. The optical density (O.D.) of each well was measured at 450 nm. The concentrations of the samples were calculated using a standard curve.

### Detection of Total and Phosphorylated Mitogen-Activated Protein Kinases in Peripheral Blood Mononuclear Cells

Quantitative analyses of Erk, JNK, and p38, as well as phosphorylated Erk (pErk), JNK (pJNK), and p38 (pp38) were conducted using the commercial electrochemiluminescent multiplex assay systems (Meso Scale Discovery, United States). Total protein and phosphoprotein were extracted from peripheral blood mononuclear cells (PBMCs) according to the instructions provided by the manufacturer. A human total MAP kinase detection kit was used to quantify Erk, JNK, p38, and a phosphorylated MAP kinase detection kit was used to determine the concentrations of pErk, pJNK, and pp38.

### Statistical Analysis

All data were analyzed by SPSS 22 (IBM Corporation, United States). The Wilcoxon signed rank test was used for comparisons between the mean levels of two independent groups. The Wilcoxon-matched pairs signed rank test was adopted to analyze the mean levels of paired samples. The Spearman rank correlation analysis was used to test the relationship between two variables. A two-sided *p* < 0.05 was considered statistically significance.

## Results

### Basic Information of the Enrolled Subjects

Subject information was listed in Tables 1, 2. The patients were male and approximately 20 years old. Compared to the contacts, patients had higher neutrophil percentages, lower lymphocyte percentages and numbers, higher monocyte percentages, and lower platelet counts (Table 1). Pneumonia patients had a longer hospital stays than other patients, and C-reactive protein (CRP) levels in pneumonia patients were higher than those in other patients (Table 2).

**TABLE 1:** Information of contacts and human adenovirus (HAdV)-infected patients.

	Contacts	Patient	
	
*n*	32	27	*p*
year [mean (SD)]	21.72 (2.43)	19.85 (2.18)	
BMI (median [IQR])	21.72 [20.75, 22.68]	22.09 [20.95, 22.81]	0.589
WBC (median [IQR])	6.19 [5.63, 7.09]	5.57 [4.79, 7.96]	0.224
Neutrophil% (median [IQR])	59.00 [53.70, 63.45]	68.50 [59.25, 76.00]	**0.005**
Neutrophil count (median [IQR])	3.71 [3.07, 4.34]	3.62 [3.09, 5.34]	0.808
Lymphocyte% (median [IQR])	33.00 [28.32, 36.08]	21.70 [16.40, 32.00]	**0.002**
Lymphocyte count (median [IQR])	2.01 [1.80, 2.38]	1.36 [0.96, 1.74]	**<0.001**
Monocyte% (median [IQR])	6.65 [5.33, 7.92]	8.50 [7.25, 9.75]	**0.004**
Monocyte count (median [IQR])	0.39 [0.32, 0.51]	0.47 [0.39, 0.58]	0.07
PLT (median [IQR])	271.00 [232.50, 294.25]	150.00 [124.50, 180.50]	**<0.001**

*WBC, white blood cell; BMI, body mass index; PLT, platelet count. The values were bold if they were < 0.05.*

**TABLE 2:** The information of human adenovirus (HAdV)-infected patients with or without pneumonia.

	Non-pneumonia	Pneumonia	
	
*n*	15	12	*P*
Days in hospital (median [IQR])	8.50 [7.00, 10.75]	11.50 [10.00, 13.25]	**0.013**
BMI (median [IQR])	22.09 [20.95, 22.57]	22.44 [21.16, 23.12]	0.574
Highest body temperature (median [IQR])	39.50 [39.45, 39.65]	40.00 [39.42, 40.00]	0.191
Fever days (median [IQR])	6.00 [6.00, 9.50]	7.00 [5.00, 9.50]	0.712
WBC (median [IQR])	5.64 [5.03, 7.12]	5.48 [4.56, 8.72]	0.884
Neutrophil% (median [IQR])	66.60 [58.95, 72.20]	72.55 [59.68, 80.48]	0.242
Neutrophil count (median [IQR])	3.76 [3.09, 4.89]	3.50 [2.88, 7.23]	0.696
Lymphocyte% (median [IQR])	23.40 [18.75, 32.00]	20.25 [13.83, 28.10]	0.294
Lymphocyte count (median [IQR])	1.43 [1.09, 1.77]	1.21 [0.93, 1.39]	0.205
Monocyte% (median [IQR])	8.70 [8.00, 9.75]	7.80 [5.82, 9.48]	0.272
Monocyte count (median [IQR])	0.50 [0.42, 0.59]	0.47 [0.36, 0.55]	0.526
PLT (median [IQR])	157.00 [145.00, 178.50]	140.50 [115.50, 180.75]	0.306
ALT (median [IQR])	12.50 [11.00, 13.75]	11.00 [9.75, 13.75]	0.338
AST (median [IQR])	25.00 [21.00, 28.75]	19.50 [17.50, 32.00]	0.455
LDH (median [IQR])	191.50 [171.75, 205.75]	172.50 [159.25, 190.00]	0.328
CRP (median [IQR])	28.20 [16.02, 38.80]	43.14 [34.08, 65.40]	**0.04**
PCT (median [IQR])	0.09 [0.06, 0.18]	0.10 [0.09, 0.15]	0.464

*ALT, alanine aminotransferase; AST, aspartate aminotransferase; LDH, lactate dehydrogenase; CRP, C-reactive protein; procalcitonin. The values were bold if they were < 0.05.*

### Human Adenovirus 7 Infection-Induced Inflammation in Patients

**FIGURE 1:**
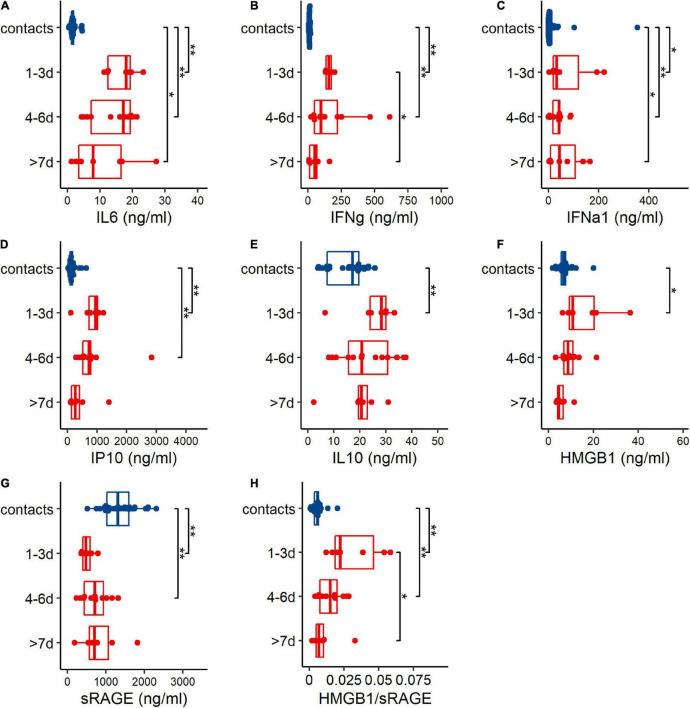
Human adenovirus 7 (HAdV-7) infection induced abnormal changes of cytokines **(A–G)** and HMGB1/sRAGE ratio **(H)** in plasma of contacts (blue points and box) and patients (red points and boxes). Based on sampling days after disease onset, patients were divided into 3 groups, 1–3 day (1–3 days post disease onset), 4–6 day (4–6 days post disease onset), and >7 day (over than 7 days after disease onset) groups. Wilcoxon test was used to compare median levels between two groups. Two side *p*-values were calculated. *p < 0.05, **p < 0.01.

To investigate the inflammatory responses after HAdV infection, inflammatory cytokines [(IL-6), (IFN-γ), (IFN-α1), interferon-inducible protein 10 (IP10)], anti-inflammatory cytokine (IL-10), (HMGB1), sRAGE, and the HMGB1/sRAGE ratio in the peripheral blood of HAdV-infected patients were evaluated, when the patients were admitted to the hospital (Figure 1). Based on the duration between admission and disease onset, patients were divided into three groups: 1–3 day (1 to 3 days after disease onset), 4–6 day (4–6 days after disease onset) and >7 day (more than 7 days after disease onset). The results showed that IL-6, IFN-γ, IFN-α1, IP10, IL-10, HMGB1, and the HMGB1/sRAGE ratio of patients in the 1–3 day group were significantly increased compared to those of the contacts. The abnormal production of IL-6, IFN-γ, IP10, IL-10, HMGB1, sRAGE, and the HMGB1/sRAGE ratio were gradually decreased along with time after disease onset. IFN-α1 concentrations in the three patient groups were comparable but higher than those of contacts.

**FIGURE 2:**
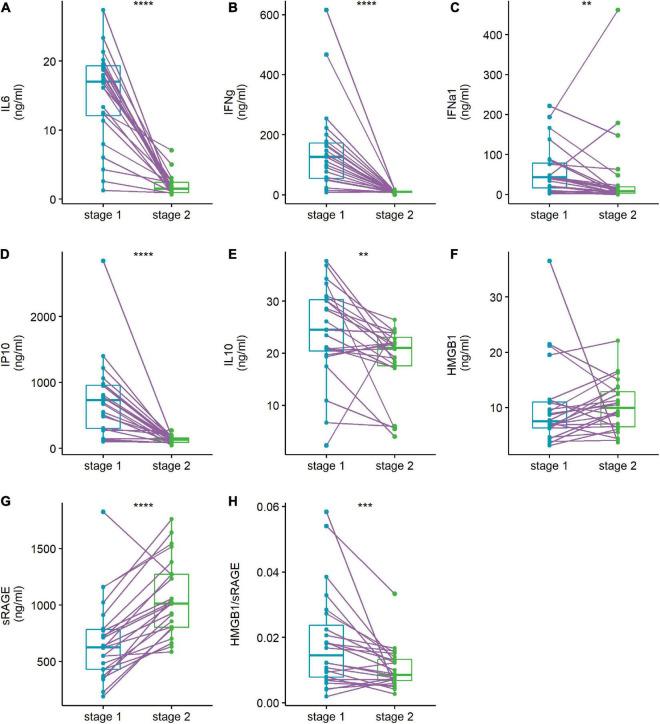
Abnormal cytokines levels **(A–G)** and HMGB1/sRAGE ratio **(H)** of patient were reversed when they discharged from hospital. Plasma cytokine levels of human adenovirus (HAdV)-infected patients were compared at admission stage (stage 1, light blue points and box) and discharge stage (stage 2, green points and box). The data points of the same patient were connected with a line. Wilcoxon-matched pairs signed rank test was used to compare median levels between two paired groups. Two side *p*-values were calculated. **p < 0.01, ***p < 0.005, ****p < 0.001.

When the patients were discharged from the hospital, IL-6, IFN-γ, IFN-α1, IP10, IL-10, and the HMGB1/sRAGE ratio were significantly reduced, and sRAGE was prominently increased (Figure 2). The concentration of HMGB1 in patients did not change significantly between the two time points.

### Reduced Soluble Receptor for Advanced Glycation End Product Levels Were Related to the Development of Pneumonia in Human Adenovirus-Infected Patients

**FIGURE 3:**
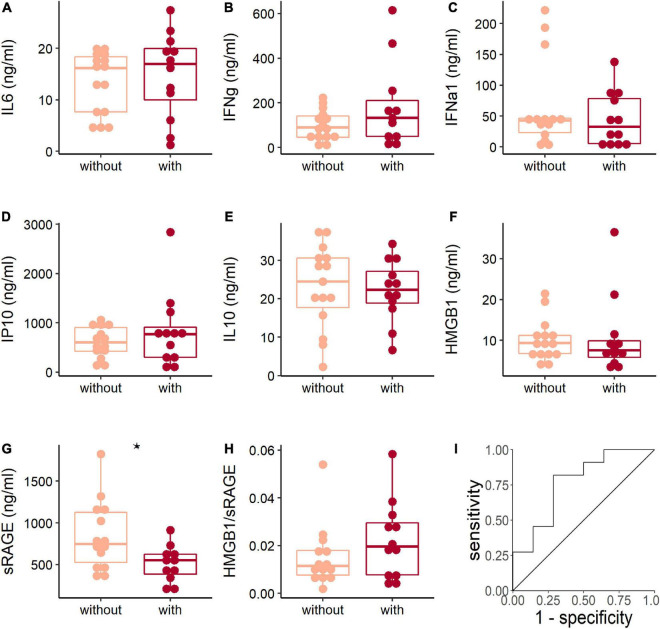
The soluble receptor for advanced glycation end product (sRAGE) concentration associated with adenoviral pneumonia. The comparison of plasma cytokines **(A–G)** and HMGB1/sRAGE ratio (H) between patients with (dark red points and box) or without (light pink points and box) pneumonia when they were hospitalized. The Wilcoxon test was used to compare median levels between two groups. Two side *p*-values were calculated. *p < 0.05. Receiver operation characteristic (ROC) analysis **(I)** of sRAGE concentration and pneumonia showed area under curve (AUC) was 0.766 (95% *CI*: 0.576–0.956) and the best threshold to distinguish patients with or without pneumonia was 633.6 ng/ml (specificity = 0.714, sensitivity = 0.818).

Pneumonia is the severe illness after respiratory HAdV infection. The results showed that the expression levels of IL-6, IFN-γ, IFN-α1, IP10, IL-10, HMGB1, and the HMGB1/sRAGE ratio were comparable between patients with or without pneumonia (Figures 3A–F,H). Meanwhile, the sRAGE level of patients with pneumonia was lower than that of patients without pneumonia (Figure 3G). Receiver operation characteristic (ROC) analysis showed that sRAGE levels could discriminate patients with or without pneumonia (Figure 3I) with a threshold of 633.6 ng/ml (sensitivity: 81.8%; specificity: 71.4%).

### Metalloprotease 9 Expression Was Related to Soluble Receptor for Advanced Glycation End Product Levels and Human Adenovirus-Induced Pneumonia

**FIGURE 4:**
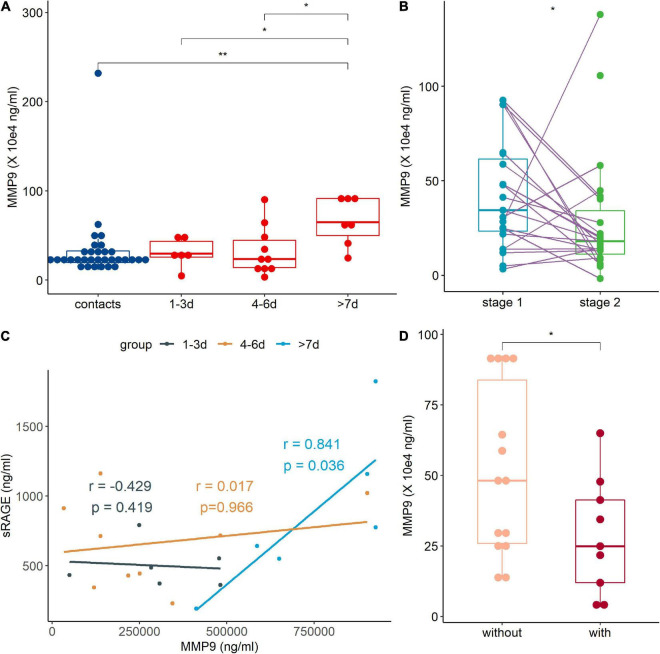
The metalloprotease 9 (MMP9) expression was regulated by human adenovirus (HAdV) infection. **(A)** Expression of MMP9 in peripheral blood of contact (blue points and box) and patients (red points and box). **(B)** Correlation analysis between MMP9 and soluble receptor for advanced glycation end product (sRAGE) of treatment naive patients. Dark, brown, and light blue represented 1–3, 4–6 day and >7 day groups, respectively. The coefficient and *p*-value were marked with the same. **(C)** Comparison of MMP9 at different stages. Stage 1 (light blue points and box) represents admission into hospital and stage 2 (green points and box) represents discharged from hospital. The data points of the same patient were connected with a line. **(D)** Comparison of MMP-9 levels between patients with (light pink points and box) or without (dark red points and box) pneumonia. r: coefficient value; *p*, *p*-value of Spearman correlation analysis. The Wilcoxon test was used to compare median levels between two groups. Wilcoxon matched pairs signed rank test was used to compare median levels between two paired groups. Two side *p*-values were calculated. *p < 0.05, **p < 0.05.

The MMP9 is an important enzymes involved in the production of sRAGE (Metz et al., 2012). We found that the MMP9 level of patients in >7 day group was higher than those of the contact group and two other patient groups (Figure 4A). Correlation analysis showed that sRAGE was positively correlated with MMP9 levels in patients in >7 day group (Figure 4B). When patients were discharged from the hospital, their MMP9 levels decreased significantly (Figure 4C). Consistent with the decrease in sRAGE in patients with pneumonia, decreased MMP9 levels were observed in pneumonia patients (Figure 4D).

The MMP9 showed a delayed response during HAdV infection, and it was elevated more than 7 days after disease onset, which might promote the resolution of HAdV-induced inflammation. The significant decrease in sRAGE in the early days after disease onset might be due to the exhaustion of sRAGE by inflammation or the decrease in other enzymes involved in sRAGE production, such as ADAM10 (Kierdorf and Fritz, 2013).

### Soluble Receptor for Advanced Glycation End Product Levels Were Associated With Mitogen-Activated Protein Kinase Pathway Activation in Immune Cells

**FIGURE 5:**
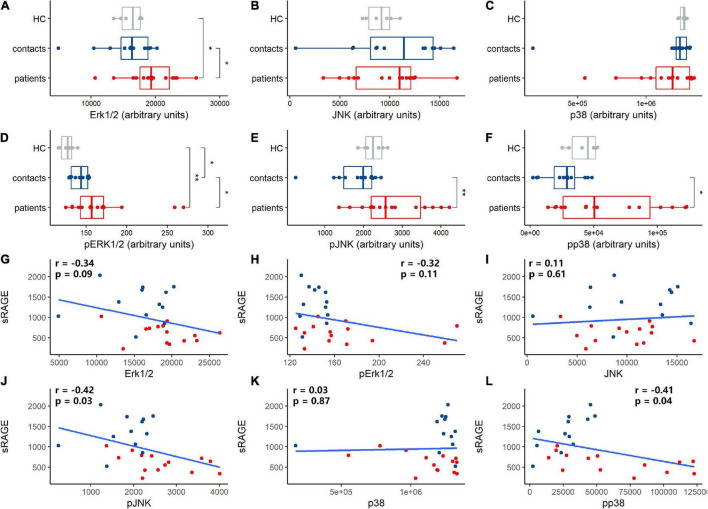
Human adenovirus (HAdV) infection-activated mitogen-activated protein kinase (MAPK) signaling, soluble receptor for advanced glycation end product (sRAGE) concentration correlated with proteins expression and phosphorylation of MAPK signaling pathway. Total Erk1/2 **(A)**, JNK **(B)** and p38 **(C)** protein and phosphate Erk1/2 **(D)**, JNK **(E)**, and p38 **(F)** protein expression in peripheral blood mononuclear cells (PBMCs) of health controls (HC, gray dots and box), contacts (blue dots and box) and patients (red dots and box) were evaluated. Correlation analysis was performed between sRAGE and proteins in MAPK signaling pathway **(G–L)** and the coefficient and *p*-value were noted in plot area as *r* and *p*, respectively. Blue dots and box represent contacts and red dots and box represent patients. pErk1/2, pJNK, and pp38 represented phosphorylated Erk1/2, JNK, and p38, respectively. The Wilcoxon test was used to compare median levels between two groups. The Spearman rank correlation analysis was adopted to analyze the association between sRAGE levels and MAPK proteins. Two side *p*-values were calculated. *p < 0.05, **p < 0.05.

Our results showed that sRAGE was negatively correlated with IL-6, IL-10, IFN-γ, and IP-10 (Supplementary Figure 1). The MAPK signaling pathway is very important in regulating many cellular processes, such as inflammation, cell differentiation, cell proliferation, metabolism, mortality, and apoptosis. In immune cells, RAGE signaling activates MAPK and results in the activation of monocytes/macrophages, dendritic cells and neutrophils and proinflammatory cytokine production (Kierdorf and Fritz, 2013; Oczypok et al., 2017). We measured the levels of the MAPK family members Erk1/2, JNK, and p38 in PBMCs and found that HAdV infection increased the total protein levels of Erk1/2 (Figure 5A), but did not influence those of JNK (Figure 5B) and p38 (Figure 5C). It induced the significant phosphorylation of Erk1/2, JNK, and p38 (Figures 5D–F). These results showed that HAdV infection activated the MAPK pathway in peripheral immune cells. The correlation analysis showed that sRAGE was not correlate with protein levels of Erk1/2 (Figure 5G), phosphorylated Erk1/2 (Figure 5H), JNK (Figure 5I) and p38 (Figure 5K). sRAGE was negatively correlated with phosphorylated JNK and p38 (Figures 5J,L).

## Discussion

Abnormal inflammatory responses contribute to the development of adenoviral pneumonia. However, the key molecule involved in the inflammatory mechanism of HAdV infection remains to be identified. In this study, we found that HAdV infection decreased sRAGE levels. sRAGE levels were associated with the production of cytokines and the development of pneumonia and correlated with MAPK activation in peripheral immune cells. Considering that sRAGE acts as a decoy receptor of RAGE. Our results suggest that RAGE/sRAGE is involved in the inflammatory responses caused by HAdV infection.

The previous publications have shown that HAdV infection induced marked inflammation in animal models and patients (Kajon et al., 2003; Lamson et al., 2020). Inflammation is necessary for initiating antiviral responses, but uncontrolled/excessive inflammation is harmful to the host and causes unexpected tissue injury. Clinical observations have shown significant inflammation in patients with severe adenoviral pneumonia induced by HAdV-7 and HAdV-55 infection (Chen et al., 2014; Sun et al., 2018). In our study, profound inflammation was indicated by the upregulation of classic inflammatory cytokines (IL-6, IFN-γ, IFN-α1, IP-10, and IL-10) in HAdV-infected patients, compared to the contacts. HMGB1 is a danger associated molecular pattern (DAMP) molecule that is considered a late stage inflammatory mediator and is linked to a range of inflammatory diseases (Ding et al., 2017). RAGE is the receptor of HMGB1. A recent animal study by Tang et al. (2018) showed that HAdV infection induced the release of HMGB1 and upregulated RAGE gene expression in lung tissue. Tang et al. (2018) suggested a relationship between RAGE function and inflammatory responses induced by HAdV in an animal model. Our results showed that in HAdV-infected patients, HMGB1 was increased in the very early days after disease onset and then decreased progressively to a level comparable to that of contacts. sRAGE, which is the extracellular segment of RAGE, can neutralize the functions of HMGB1. We found a significant increase in the HMGB1/sRAGE ratio in HAdV-infected patients. This result suggested that HMGB1 was relatively abundant to induce RAGE activation. Then, we found that sRAGE was different between patients with and without pneumonia, which suggested that sRAGE might be involved in HAdV-induced lung inflammatory responses.

Peripheral immune cell activation is one of the important hallmarks of HAdV infection (Chen et al., 2014). MAPKs are important for immune activation and inflammatory responses (Arthur and Ley, 2013). We found elevated phosphorylation of Erk1/2, JNK, and p38 in the PBMCs of HAdV-infected patients, which indicated significant immune cell activation. MAPKs are activated by RAGE signaling. Our results showed that sRAGE levels were negatively correlated with phosphorylated JNK and p38. This result suggested that sRAGE inhibited MAPK activation by inhibiting RAGE.

We also examined the factors leading to the changes in sRAGE concentrations. MMP9 is one of the key enzymes involved in the production of sRAGE. MMP9-mediated sRAGE production inhibited the RAGE/NF-κB activation and consequently reduced the severity of pulmonary edema, inflammation, and oxidative stress (Zhang et al., 2021). During SARS-CoV-2 infection, patients showed increased expression of MMP9 compared to controls (Gelzo et al., 2022). In our study, MMP9 was not very sensitive to HAdV infection in the early days (less than 6 days), and patient MMP9 was only elevated more than 7 days after disease onset. This result could partially explain the progressive increase in sRAGE levels in patients with the duration of disease. Consistent with the low sRAGE levels in pneumonia patients, MMP9 levels in patients with pneumonia were lower than those in patients without pneumonia. This finding suggested that the reduced sRAGE production in pneumonia patients might be due to the reduced MMP9 expression, which was insufficient to ameliorate lung inflammation induced by HAdV infection.

The current study was an observational clinical study, and association among sRAGE, adenoviral pneumonia, and inflammatory responses was observed in HAdV-infected patients. The mean age of the study population was approximately 20 years old, and all subjects were male. Elderly individuals, children, and females are also vulnerable to HAdV infection and further study focusing on those populations could provide more evidence to support the role of sRAGE in HAdV-induced inflammation.

## Conclusion

Our data suggest that RAGE/sRAGE is involved in the inflammatory responses induced by HAdV infection in patients.

## Data Availability Statement

The raw data supporting the conclusions of this article will be made available by the authors, without undue reservation.

## Ethics Statement

The studies involving human participants were reviewed and approved by the Beijing 302 Hospital Research Ethnics Committee (2018032D). The patients/participants provided their written informed consent to participate in this study.

## Author Contributions

WX: conceptualization, formal analysis, investigation, writing – original draft preparation, and funding acquisition. C-JW: conceptualization and writing – original draft preparation. Y-MJ: conceptualization and methodology. X-LM and LH: investigation and formal analysis. E-QQ, BT, and PZ: investigation. L-FW: conceptualization. W-WC: conceptualization, supervision, writing – reviewing and editing, project administration, and funding acquisition. All authors contributed to the article and approved the submitted version.

## Conflict of Interest

The authors declare that the research was conducted in the absence of any commercial or financial relationships that could be construed as a potential conflict of interest.

## Publisher’s Note

All claims expressed in this article are solely those of the authors and do not necessarily represent those of their affiliated organizations, or those of the publisher, the editors and the reviewers. Any product that may be evaluated in this article, or claim that may be made by its manufacturer, is not guaranteed or endorsed by the publisher.
