# Supplementary material for: Soluble Receptor for Advanced Glycation End Product Is Involved in the Inflammatory Response of Human Adenovirus-Infected Patients
Source: Front Microbiol. 2022 Jul 7;13:923215. doi: 10.3389/fmicb.2022.923215 (PMC9301492; doi:10.3389/fmicb.2022.923215)
Supplement: Supplementary file 1 [file Data_Sheet_1.docx]

Supplementary Material


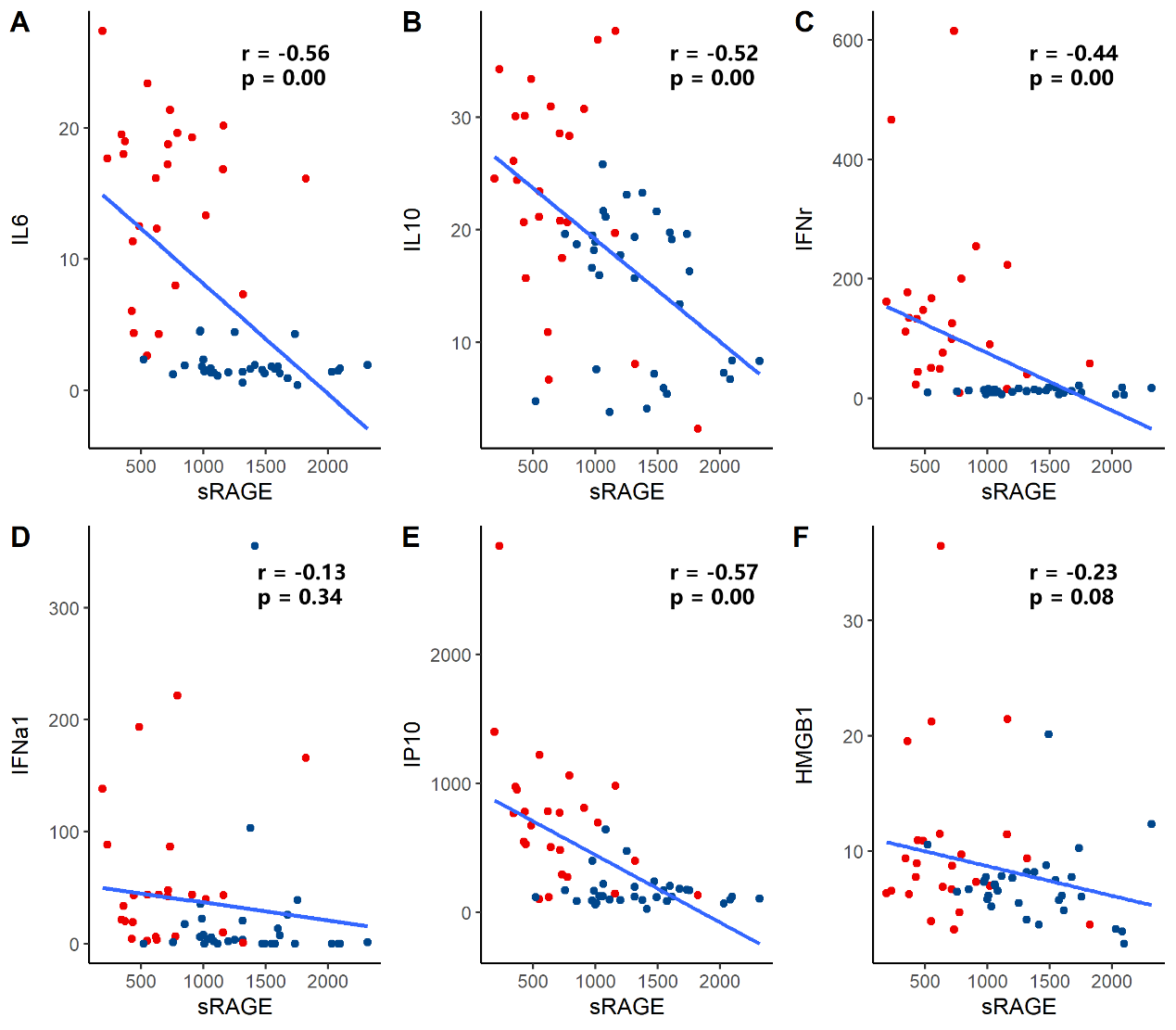


**Supplementary Figure 1:** sRAGE levels correlated with proinflammatory cytokines production. Red dots represented patients and blue dots represented contacts. Coefficient and p values were noted as r and p in plot area. sRAGE: soluble receptor for advanced glycation end products; IL: interleukin; IFNr: interferon gamma; IFNa1:interferon alpha 1; IP10: IFN gamma inducible protein 10; HMGB1: high mobility group protein B 1.
